# Differences between Female and Male Inmates in Animal Assisted Therapy (AAT) in Austria: Do We Need Treatment Programs Specific to the Needs of Females in AAT?

**DOI:** 10.3390/ani10020244

**Published:** 2020-02-04

**Authors:** Birgit U. Stetina, Christine Krouzecky, Lisa Emmett, Armin Klaps, Nora Ruck, Zuzana Kovacovsky, Anastasiya Bunina, Jan Aden

**Affiliations:** 1 Psychological Outpatient Clinic, Sigmund Freud University Vienna, 1020 Vienna, Austria; christine.krouzecky@sfu.ac.at (C.K.); lisa.emmett@sfu.ac.at (L.E.); armin.klaps@sfu.ac.at (A.K.); zuzana.kovacovsky@sfu.ac.at (Z.K.); 2 Faculty of Psychology, Sigmund Freud University Vienna, 1020 Vienna, Austria; nora.ruck@sfu.ac.at (N.R.); anastasiya.bunina@sfu.ac.at (A.B.); jan.aden@sfu.ac.at (J.A.)

**Keywords:** gender-differences, prison, animal-assisted therapy, gender-sensitive treatment, criminal offenders, substance dependency syndrome

## Abstract

**Simple Summary:**

So far, many studies in the field of animal assisted therapies (AAT) show promising results in several areas for male and female participants. A newer study has found auspicious results for the target group of female inmates in 2013, which prompted our literature research and an additional gender and sex related evaluation of especially selected intervention data collected over a time period of ten years in specialized institutions with criminal offenders suffering from substance dependence syndrome. We found in our analysis that although male and female participants benefit both from the dog assisted group therapy (MTI), female drug dependent criminal offenders benefit less, especially regarding skills connected with emotions. Although practice and research in the area of AAT is booming and most studies promise positive results for a large variety of people, research has to focus on who benefits from which kind of intervention, what intensities and numbers of sessions are needed, and what prerequisites need to be met to allow benefits for all beings involved.

**Abstract:**

With the growth of female inmates worldwide, research regarding specific treatment of these has become more important. Although new programs have been started, the lack of scientific results is startling. The goal of the current study was to identify differences between participants from the men’s and women’s section in a specialized prison for criminal offenders suffering from substance dependence syndrome regarding the effects of dog-assisted group therapy. Therefore, 81 incarcerated participants (50 male, 31 female) took part in a dog-assisted group therapy targeting socio-emotional competencies. Self-report questionnaires to measure self-concept (SDQ-III), emotional status (EMI-B) and emotional competencies (SEE) were employed. Statistical analysis included General Linear Model (GLM) procedures and η^2^ as concurrent effect size measure. Results demonstrate that participants from the women’s ward tend to benefit significantly less from the dog-assisted group therapy in most measured areas than men, especially in terms of their emotional status (e.g., aggressiveness) and emotional competencies (e.g., emotion regulation). Treatment programs specific to the needs of women might be a future challenge for practitioners and researchers in AAT.

## 1. Introduction

Only 6.9% of the global prison population are girls or women. Nevertheless, the proportion of female inmates is increasing much faster than the population of incarcerated men [1]. In fact, since 2000 the worldwide number of female inmates grew 50.2%, while the number of male inmates only grew 18.1% [2]. In Austria, there are 8.497 individuals in prison, 623 of them are female. This corresponds to a share of 6.74% [3]. Relevant factors contributing to this increase are changes in sentencing laws, as well as the war on drugs. This has brought many women to the attention of the criminal justice system [4]. Which is not only a US phenomenon, but a worldwide result of similar drug strategies.

The higher number of incarcerated women, as well as the women’s emancipation movement altogether, led to a more intensive scientific examination of the specific needs of women in jail since the 1970ies [5]. Research focusing on differences between male and female inmates shows significant disparities in factors related to substance dependence [4]. For example, substance use and dependency problems have been found to be more prevalent among female inmates, than among their male counterparts [6]. Studies also determine that substance-abusing women are more likely to have history of sexual and physical abuse, tend to meet the criteria of a coexisting psychiatric disorder and show a lower self-esteem than incarcerated drug using men [4,6]. Finally, the focus regarding treatment addressing the specific needs of females and males should be on aspects such as maternal mental health, the development of secure parent–infant attachments, safe environment training and universal relationship education [7]. 

Even though women are the fastest growing segment of the prison population and research regarding gender-sensitive approaches have shown significant differences between men and women, the penal system is still mostly male-oriented, and female-specific programs are rarely offered [8]. Therefore, the omitted aspects that need to be reformed include treatment oriented toward women’s needs and aftercare programs with regard to resocialization after release, as well as the scientific review [9].

Nevertheless, several prisons implement various kinds of therapeutic interventions, ranging from basic individual therapy to group therapy, as well as skills training [10]. A specific form of skills training offered in a limited number of institutions is animal-assisted therapy (AAT). Research in the context of AAT increasingly emphasizes the bio-psycho-social effects of human-animal bonds on human well-being. Overall, a large number of studies concerning this topic show unique benefits of the presence of and the relationship with animals on human mental and physical health. In this context, positive effects could not only be determined within long term interactions, such as dog ownership, but also in short term interactions regarding therapy dogs [11]. Studies concentrating on animal-assisted therapy indicated therapeutic and rehabilitative benefits for psychological and medical concerns across a variety of settings, such as inpatient units, hospitals and educational institutions [12]. This form of therapy is defined as the use of the human–animal bond through strategically selected and goal-directed intervention, in which the animal is an integrated and primary component of the treatment process [13]. Research focusing on AAT with inmates reported several positive results, especially regarding the development of social and emotional skills [10]. Moreover, studies suggest differences between male and female participants regarding social and emotional competences [14]. In this context, female inmates demonstrate higher competence in both areas, which in turn may represent a protecting factor against delinquency [15].

As aforementioned, there is evidence that female inmates have specific needs within therapeutic treatments, which are not sufficiently considered throughout intervention programs because they are mostly designed for men. Additionally, female inmates also differ from the male ones in regard to social and emotional competences [16]. Therefore, the present study tries to examine these aspects using the example of animal-assisted therapy (dog assisted group therapy) and tries to investigate the effectiveness in female and male inmates. In order to verify, if female and male inmates differ in terms of their benefit from AAT, a specific concept of animal assisted interventions, named multi-professional animal-assisted intervention (MTI), was conducted. 

The MTI concept focuses on the development of social and emotional competences through training with a focus on the specific forms of canine interaction (e.g., nonverbal communication forms of dogs among dogs), needs of the animal, and interaction with the dog. The concept has been used in a variety of prison settings and specialized forensic institutions. The presented data set is one part from a bigger data set from a specialized institution for criminal offenders with substance dependence syndrome. MTI has been evaluated in the institution with several groups of female and male inmates over a ten-year period using an RCT design (e.g., [17]). As the present study focused on differences within participants from the women’s and men’s females and males. Therefore, control groups and other therapy groups were not included in the current paper.

## 2. Materials and Methods 

Ethical approval was granted by the “Ethics Commission of the Faculty Psychotherapy Science and Faculty of Psychology” of the Sigmund Freud University Vienna (NBGQFDXMAFPECW87490).

### 2.1. Program Description

The evaluated and manualized dog assisted group therapy program MTI [18], which stands for the German term Multiprofesionelle Tiergestützte Intervention and means multiprofessional animal assisted intervention, is a resource oriented skill training that consists of ten or twelve dog-assisted group sessions, which take place once a week for one hour with two professionals (multiprofessional: psychologist and pedagogue; within this study), at least one animal co-therapist (within this study a female black labrador retriever) and eight to ten participants. MTI, as dog assisted group therapy, is a competence and communication training that aims to enhance the social and emotional skills of the participants learning through the interaction with the dog based on social and emotional skills that humans can learn from canines for socio-emotional interactions (e.g., the variety of canine reactions in dangerous situations) The multi-professional animal-assisted intervention concept is based on a bio-psycho-social understanding of health. It includes a flexible approach using predefined modules and their components from a manual [18] to increase transferability, which can be mixed and varied according to the training needs of the participant group. The basic character is the appreciative interaction with the dog. Due to the common responsibility of the group for the well-being of the dog (under supervision of the trainers of course and with very clear rules and boundaries), individual emotional control can be learned, and social competencies can be strengthened [18]. One example for learning social and emotional competences would be the respectful atmosphere created, during the training. The so called “violence-free togetherness” enables participants to experience respect and to learn the meaning of appropriate and responsible interaction with humans and animals [18]. In this context, the equal position of the animal as trainer in the group play an essential role and have to be respected. They already need consideration (1) in the education of the dog, (2) in the performed work, (3) in the preparation of the dog for the work, and (4) in the assessment of quality of the work of the dog [19] to prepare an animal for the role as trainer in the group setting of MTI.

For the target group of participants from this forensic institution focusing on the treatment of substance dependent criminal offenders, the ten-week program has been chosen and eight inmates have taken part in the weekly dog-assisted group for one hour per session. Two training rounds were carried out per year, by two female trainers. 

### 2.2. Study Design and Participants

In order to analyse differences regarding the effectiveness of dog-assisted interventions in the emotional and social competences of inmates, participants from the women’s and men’s ward were compared concerning standardized measurement results and demographic data. Using a pre-post-design, three different self-report questionnaires (Scales for Experiencing Emotions; Emotionality Inventory as a Measure of Well-being; Self Description Questionnaire III) were completed by the inmates before and after ten weeks of dog-assisted therapy. In regards to the standardization process, only criminal offenders with the ICD 10 diagnosis “Psychological and behavioural disorders caused by multiple substance use and consumption of other psychotropic substances: dependence syndrome” [20] in a closed prison setting (institution with focus on the treatment of substance dependent criminal offenders) have been included in the statistical analysis. The described institution houses female and male inmates in separate divisions.

Additionally, only data from those inmates who worked with the same therapeutic team (two female human professionals and the non-human trainer, Emily, a female black labrador retriever) were used. In total, 81 fully completed data sets that have been collected over 10 years were included in the calculations (n = 81). The sample consists of 33.3% women (n = 31) and 66.7% men (n = 50), with an average age of 29 years (M = 29.3, SD = 7.24). 

### 2.3. Instruments

#### 2.3.1. Demographic Data

Collected data included age and gender (based on the classifications in female and male division, which is based on official gender identity) and a variety of questionnaires testing social and emotional status and competencies. These data were considered possible influencing factors on the results regarding the effects of dog- assisted interventions (MTI). 

#### 2.3.2. Scales for Experiencing Emotions (SEE)

The Scales for Experiencing Emotions were used for measuring how people perceive, evaluate and deal with their own emotions [21]. The questionnaire consists of 42 items, including the subscales “accepting own emotions”, “emotional flooding”, “lack of emotions”, “somatic representation”, “imaginative representation”, “emotional regulation” and “self-control”. The response alternatives have a range between 1 point and 5 points (from totally disagree to totally agree). The internal consistency amounts to α = 0.86. 

Example statement: “I am not ashamed of my feelings”.

#### 2.3.3. Emotionality Inventory as a Measure of Well-being (EMI-B)

The Emotionality Inventory as a Measure of Well-being was used to operationalize the changes within the period of the animal-assisted interventions, regarding the emotional dimension of “well-being” [22]. The scale contains 70 bipolar arranged items, asking about the well-being of the last 7 days, which can be assessed in a six-fold gradation possibility. The items include the following seven subscales: “anxious versus free from fear”, “depressive versus happy”, “tired versus dynamic”, “aggressive versus calm”, “inhibited versus spontaneous”, “lonely versus secure” and “imbalanced feeling versus well-being”. The internal consistency amounts to α = 0.98. 

Example item: “tense versus relaxed”. 

#### 2.3.4. Self Description Questionnaire III (SDQ-III) 

The Self Description Questionnaire III was used for measuring the multidimensional self-concept of the sample [23]. It measures thirteen different dimensions of self-concept and consists of 136 Items that can be answered on an eight-point rating scale (from definitely false to definitely true) including the subscales “math”, “verbal”, academic”, “problem solving”, “physical ability”, “same sex peer relations”, “opposite sex peer relations”, “parent relation”, “spiritual values/religion”, “honesty/trustworthiness”, “emotional stability”, “general esteem” and “physical appearance”. The internal consistency amounts to α = 0.89.

Example statement: “I am usually pretty calm and relaxed”.

### 2.4. Statistical Analysis

A one-way repeated measures ANOVA (Analysis of Variance) was used to compare the group means of each questionnaire at the two test times. This method was chosen in order to analyse the inner-subject differences in each questionnaire, before and after the dog-assisted intervention. Moreover, a one-way ANOVA for independent samples was used for analysing pre-post differences among males and females. For all analysis, the significance level was set at *p* ≤ 0.05 and η^2^ was used as a concurrent effect size measure (effect size = denotes the size of a statistical effect). In addition, a Bonferroni correction was performed to neutralize the Type I error. Analyses were computed with SPSS 24.0.

## 3. Results

### 3.1. Differences between Male and Female Participants Regarding the Scales for Experiencing Emotions (SEE)

**Figure 1:**
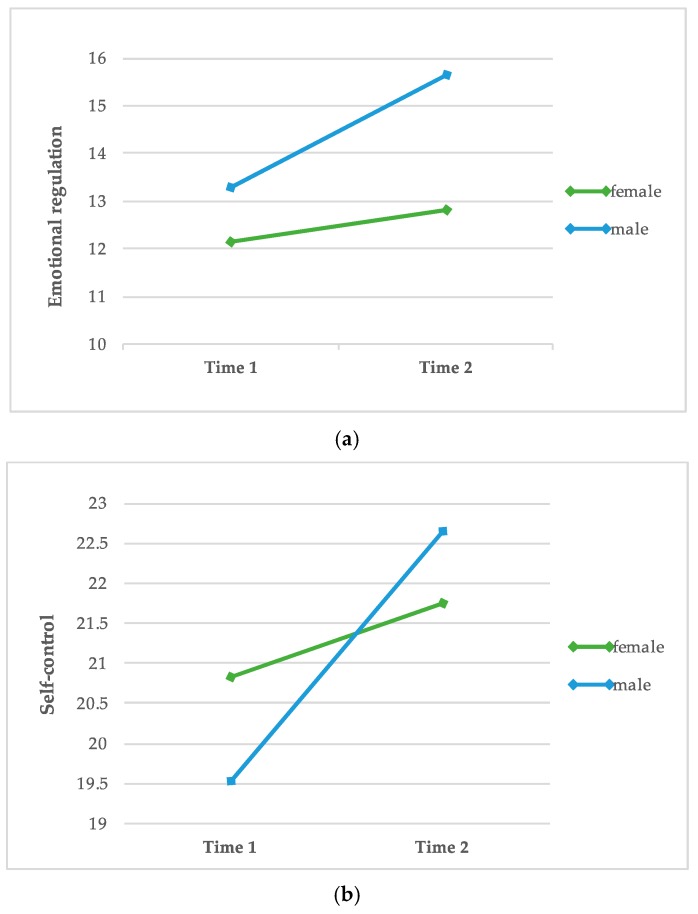
Differences between male and female participants regarding the subscales “emotional regulation” (**a**) and “self-control” (**b**).

In order to test in which areas men and women differ regarding the SEE at both test dates, a one-way repeated measures ANOVA was conducted. Table 1 and Table 2 represent the results of the calculation. Additionally, Figure 1 shows the strongest changes of men and women regarding to the subscales of the SEE (measured by the strength of effect size). 

Results demonstrate that male inmates show a bigger change in the intended direction of dog-assisted intervention than female inmates in five out of seven subscales. In this context, positive effects can be found in the subscales “accepting emotions”, “emotional regulation”, “self-control”, “emotional flooding” and “lack of emotions”. Regarding the subscale “emotional flooding”, data additionally indicate a significant positive development in female inmates. 

### 3.2. Differences between Male and Female Participants Regarding the Emotionality Inventory As a Measure of Well-being (EMI-B)

**Figure 2:**
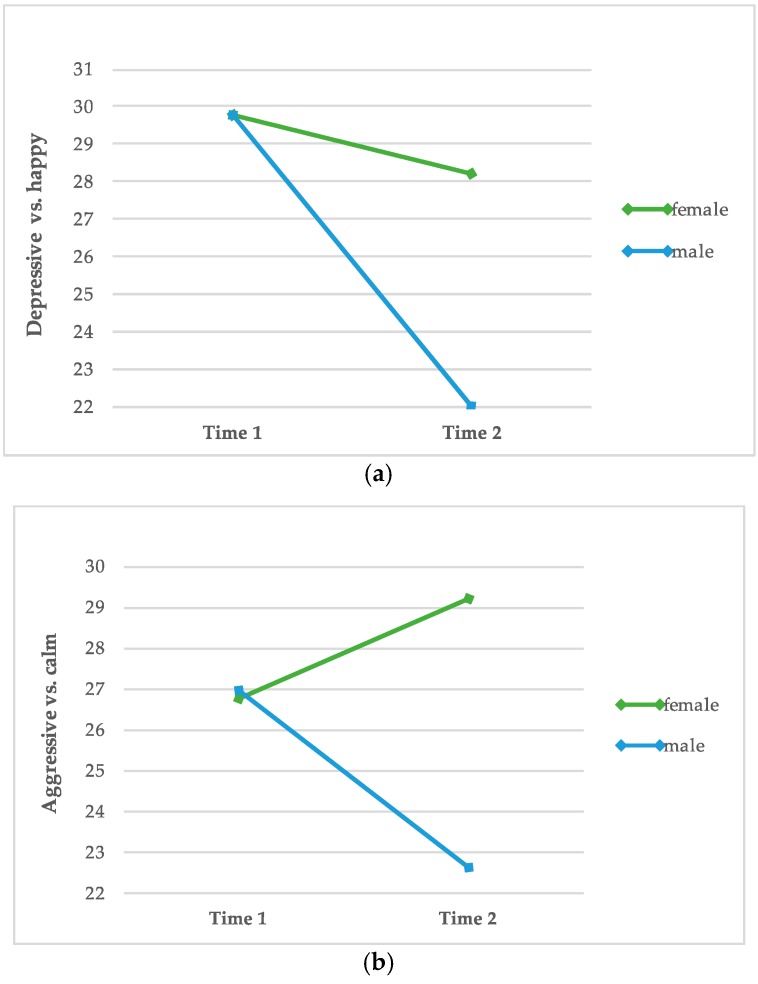

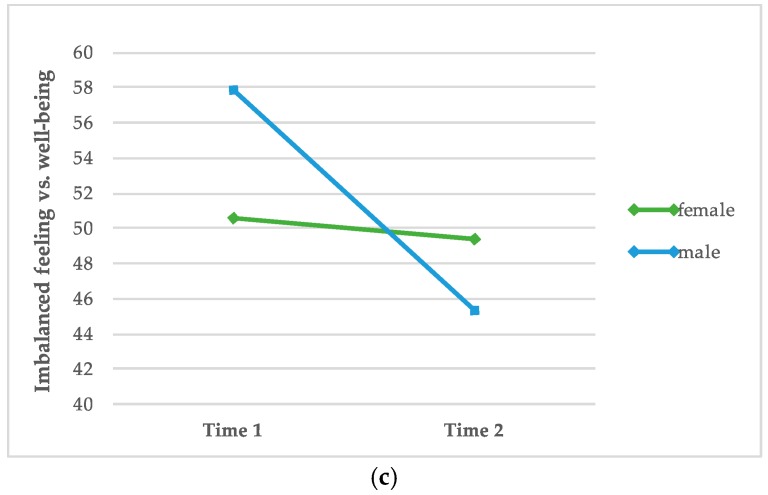
Differences between male (n = 36) and female (n = 27) participants regarding the subscales “depressive versus happy” (**a**), “aggressive versus calm” (**b**) and “imbalanced feeling versus well-being” (**c**).

In order to verify in which areas female and male inmates differ regarding the EMI-B at both test dates, a one-way repeated measures ANOVA was conducted. Table 3 and Table 4 represent the results of the evaluation. In addition, Figure 2 shows the strongest changes of men and women regarding to the subscales of the EMI-B (measured by the strength of effect size).

Results of all seven subscales indicate a significant and greater reduction of emotional suffering in male inmates, than in female inmates. Moreover, data show a significant increase, regarding the subscale “aggressive versus calm” in female inmates, as well as a significant reduction of the subscale “lonely versus secure”. 

### 3.3. Differences between Male and Female Participants Regarding Self Description Questionnaire III (SDQ-III)

**Figure 3:**
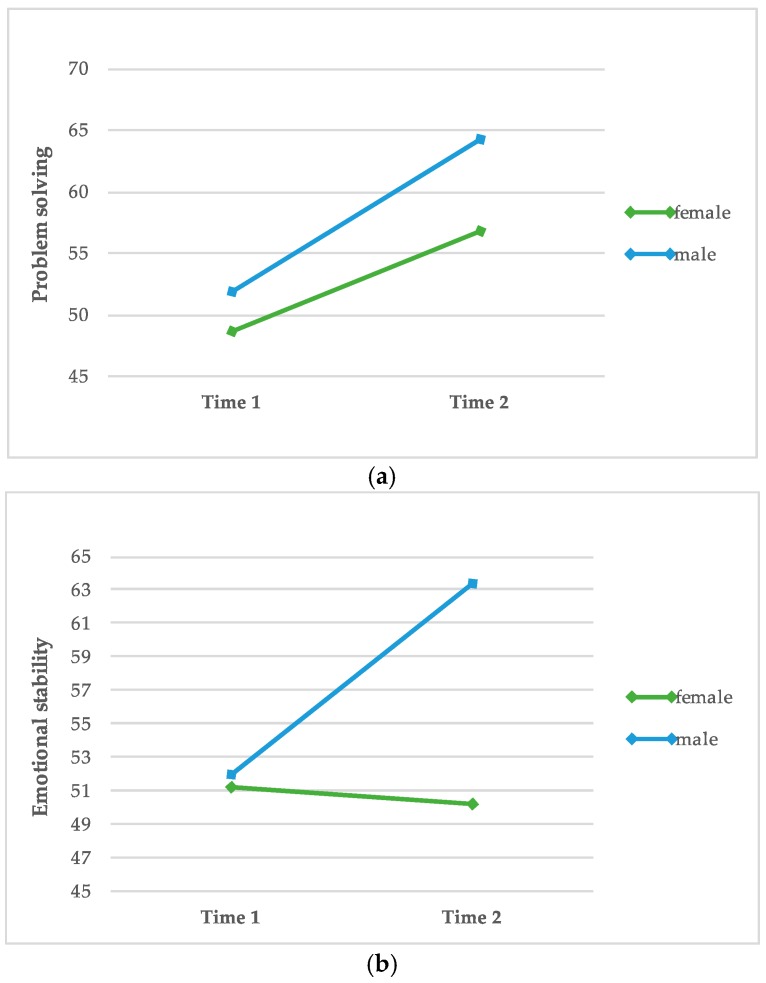

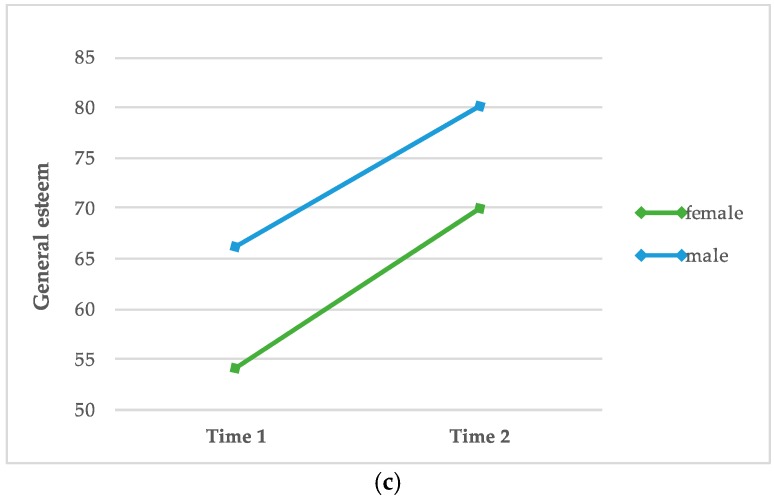
Differences between male (n = 49) and female (n = 31) participants regarding the subscales “problem solving” (**a**), “emotional stability” (**b**) and “general esteem” (**c**).

In order to test in which areas men and women differ regarding the SDQ-III at both test dates, a one-way repeated measures ANOVA was computed. Table 5 and Table 6 represent the results of the calculation. Additionally, Figure 3 shows the strongest changes of men and women regarding the subscales of the SEE (measured by the strength of effect size).

Data demonstrate a significant increase in seven out of thirteen subscales in the desired therapeutic direction (“verbal”, “problem solving”, “physical ability”, “same sex peer relations”, “opposite sex peer relations”, “honesty/trustworthiness” and “general esteem”). Moreover, results indicate a significant reduction in the subscale “spiritual values/religion”. 

Results show a significant increase in nine out of thirteen subscales in the desired therapeutic direction (“math”, “verbal”, “academic”, problem solving”, same sex peer relations”, opposite sex peer relations”, “honesty/trustworthiness”, “emotional stability” and “general esteem”). 

Summarizing the data of Table 5 and Table 6, regarding the mean differences in male and female inmates, the subscales of the SDQ III indicate a bigger change in the intended direction of dog-assisted intervention in male rather than female inmates. 

## 4. Discussion

The findings of the presented dataset show an increase in emotional and social competences of male and female inmates during the period of the MTI training when comparing pre and post data. Nevertheless, stronger significant improvements were found in male inmates. In this context, a positive development concerning emotional regulation, self-control, emotional stability and problem solving could be found. Additionally, male inmates showed a significantly stronger increase regarding their well-being.

Although emotional and social competences of female inmates also improved during the period of the MTI training, present data indicate a greater effect of the dog-assisted interventions in male inmates. A variety of potential reasons has to be considered when discussing the results. Possible explanations include the better health condition of the male participants and less associated stress factors compared to the female inmates. In this context, results demonstrate that in almost all questionnaires, women generally reported a lower level of competences than men. This might be explained through consideration of the disadvantaged background women who enter prison often come from [24]. Additionally, there is evidence that a high number of incarcerated women have experienced sexual and physical abuse prior to their detention and therefore are at higher risk to experience physical problems as for example be infected with sexually transmitted diseases [25]. Therefore, the initial status is already on a lower level in females than males. A comparison of that initial status shows that, in the described population of vulnerable participants, the participants scores from the female division demonstrate a higher level of vulnerability in comparison with their male counterparts, which has only been considered in a limited number of studies. In the presented data collection and evaluation, the resource-oriented focus led to a primary analysis in effectiveness of the program. Initially, the potential role of the program in the rehabilitation process of the participants was the focus and the resource-oriented viewpoint of the training was supported by the positive outcome results for both divisions. Only the additional calculations showed the differences between the participants which is a good example for the extra effort that needs to be made to identify the needs of all groups involved as participants. This extra step is more difficult, the reactions to the results are at least unclear as the whole program might be questioned and the concurrent discussion poses a risk when showing differences in effectivity. Although not an excuse, this is an explanation for the missing evaluation of effectivity of different subgroups of populations in similar studies.

In addition, an increase in the female inmate population can be observed but there is no report of an increase in treatment programs specific to the needs of females [8]. This aspect seems relevant because of significant differences between men and women regarding biological fundamentals and social needs. Female inmates for example differ especially in their needs in terms of medical care. They depend on health care services in regard to menstruation, pregnancy, childbirth etc. and these services are obviously more complicated to offer in prison. Bearing a child away from home without the support of family or friends seems to be a stressful experience for these women [26]. It is hard to go through a pregnancy, particularly when also suffering from mental illness, something that is highly prevalent with regards to female inmates [6]. Finally, the incarceration of mothers represents a higher damage for family systems, as women are more likely to be bestowed with the responsibility for caring for the children [25]. Therefore, this aspect can trigger an intense feeling of shame and distress for the imprisoned women because they are not able to fulfill their roles as mothers and properly care for their children, which can also lead to experiencing rejection by their families or by society [27]. 

Results also demonstrate that the female inmates’ aggressiveness significantly increased during the period of the MTI training. Since there is evidence that women are more likely to suppress or engage in predominantly indirect aggression [28], it might be assumed that by promoting the perception of emotions, the access of participating female inmates to negative emotions may have also been strengthened. Research concerning this topic demonstrate that suppression of aggression is highly correlated with low levels of self-esteem [29]. This aspect leads to the assumption that the increased aggressiveness of female inmates might be the effect of a development in the self-esteem, which could also be determined within the present study. 

Overall, the results of the present dataset indicate that AAT might be more appropriate for male inmates than female inmates. This result this is also consistent with other studies, which indicate no significant improvement in the mental health of female inmates, during AAT training [9]. Taking all results into consideration, it however is presented that female inmates in our study show significant increases in the area of self-esteem. Based on these findings, it can therefore be assumed that the perception of emotions in female inmates has been improved. Results, such as the increased aggression level, nevertheless indicate that female inmates still have difficulties with the handling of emotions, which might further be compounded by the increased self-esteem. This interpretation is also supported by results regarding the emotional stability, which decreased during the period of the MTI training with the female inmates. It could be deduced from this aspect that female inmates could have learned better to deal with their emotions, using the longer version of the program [30].

## 5. Conclusions

In summary, there are several relevant facts that should be taken into consideration. Animal assisted therapy shows promising results in prison, tested in this study with the dog-assisted group therapy program MTI focusing on social and emotional competences. Female and male participants show significant changes into a positive direction, but females benefit especially in the area of self-esteem and males benefit in a larger variety of socio-emotional competences. Therefore, it seems the field of AAT or AAI in general must consider programs designed for the specific needs of females, especially in prison. When implementing such a female sensitive treatment or intervention within the prison setting it seems relevant to focus on aspects such as relationship trainings or the development of secure attachment styles. Therefore, AAT seems to be especially suitable as the development of a relationship with another species represents an opportunity for building safe attachments for those who have lost their trust in humans for example because of experienced abuse which its common among female inmates [31]. Lastly, AAT, when based on respectful interactions between all interacting counterparts of all species may represent an integral component of an over-all concept for female inmates.

## 6. Limitations

The presented study did only analyse data from participants in the Austrian penitentiary system therefore generalisation is limited to similar systems. AAT as part of a general treatment approach can only be as good as the general concept which includes a certain program. Therefore, the results need to be perceived and discussed carefully. However, in light of the inequality in treatment of men and women, the growing knowledge of diverse needs in a variety of target groups might be seen as a potential trigger for future research. In addition to that, future research should focus on the needs of participants, including all LGBTQ categories. Furthermore, an international multicentre study including different systems would provide the necessary insight for generalisation.

In discussing the presented results, it is obvious that a more flexible research design, in the sense of more sessions when needed without concurrent administrative limitations, would have allowed more insights into the dynamics of change in male and female participants. Therefore, the study did not analyse the effects of the intervention with regards to the number of sessions. Also, the drop-out rate of participants must be considered in the future, which was caused due to relocations, early releases etc. This circumstance has led to a relatively small number of participants, so the results should be interpreted with caution. One might argue that the longer form of MTI could have led to better results for the female participants. Therefore, a future study which includes the mentioned flexible approach with an even unlimited amount of sessions and several time points for testing to find the “ideal number” for change would add valuable insights for one of the major future challenges in AAT research: the question of “dosage” in the sense of needed contacts to start change or reach a predefined behavioural or cognitive goal. Other limiting factors could be the fact that both trainers in this study were women, what might has affected the outcome in the different gender groups and secondly that they, as well as the therapy dog, possibly changed in some way during the period of the ten years. Furthermore, the internal validity of this design is limited, as no control group was included in the calculations. These aspects have not been included in the evaluation and should be considered in future studies. 

## Figures and Tables

**Table 1:** Mean differences in females regarding the subscales of the SEE before and after dog-assisted intervention.

Subscales SEE	Time 1		Time 2		F	df 1	df 2	*p*	η²
	M	SD	M	SD					
accepting own emotions *	22.30	3.56	23.23	2.69	1.293	1	30	0.264	0.041
emotional flooding	21.93	3.91	20.22	3.81	4.97	1	30	0.033	0.142
lack of emotions *	13.35	2.81	13.29	2.28	0.015	1	30	0.903	0.001
somatic representation	24.77	6.49	27.03	4.78	3.25	1	30	0.082	0.098
imaginative representation	17.90	4.64	17.32	4.93	0.526	1	30	0.474	0.017
emotional regulation *	12.16	3.01	12.80	2.7	3.250	1	30	0.081	0.098
self-control *	20.83	3.66	21.74	4.48	3.125	1	30	0.259	0.042

* significant difference between female and male participants in pre-post difference according to GLM (ANOVA)—details see Appendix A.

**Table 2:** Mean differences in males regarding the subscales of the SEE before and after dog-assisted intervention.

Subscales SEE	Time 1		Time 2		F	df 1	df 2	*p*	η²
	M	SD	M	SD					
accepting own emotions *	21.46	4.69	24.70	3.8	29.26	1	49	0.001	0.374
emotional flooding	21.36	5.84	19.34	5.03	9.567	1	49	0.003	0.163
lack of emotions *	13.82	3.97	12.20	3.32	17.2	1	49	0.001	0.260
somatic representation	25.20	5.98	26.30	5.45	1.67	1	49	0.202	0.033
imaginative representation	16.40	4.82	16.98	4.68	1.025	1	49	0.316	0.020
emotional regulation *	13.30	3.11	15.66	2.37	41.66	1	49	0.001	0.460
self-control *	19.54	4.36	22.64	3.82	31.041	1	49	0.001	0.388

* significant difference between female and male participants in pre-post difference according to GLM (ANOVA)—details see Appendix A.

**Table 3:** Mean differences in females regarding the subscales of the EMI-B before and after dog-assisted intervention.

Subscales EMI-B	Time 1		Time 2		F	df 1	df 2	*p*	η²
	M	SD	M	SD					
anxious versus free from fear *	64.44	9.48	65.11	6.86	0.319	1	26	0.577	0.012
depressive versus happy *	29.78	4.37	28.22	4.39	3.288	1	26	0.081	0.112
tired versus dynamic *	29.56	4.13	30.00	2.03	0.443	1	26	0.511	0.017
aggressive versus calm *	26.78	5.01	29.22	2.87	8.837	1	26	0.006	0.254
inhibited versus spontaneous	32.11	5.67	32.44	3.15	0.144	1	26	0.707	0.006
lonely versus secure	30.67	3.75	29.11	2.37	6.723	1	26	0.015	0.205
imbalanced feeling versus well-being *	50.56	11.12	49.33	14.4	0.322	1	26	0.575	0.012

* significant difference between female and male participants in pre-post difference according to GLM (ANOVA)—details see Appendix A.

**Table 4:** Mean differences in males regarding the subscales of the EMI-B before and after dog-assisted intervention.

Subscales EMI-B	Time 1		Time 2		F	df 1	df 2	*p*	η²
	M	SD	M	SD					
anxious versus free from fear *	57.61	12.97	49.16	12.38	13.546	1	35	0.001	0.279
depressive versus happy *	29.78	8.04	22.00	7.27	39.902	1	35	0.001	0.533
tired versus dynamic *	29.00	7.65	24.36	6.84	11.03	1	35	0.002	0.240
aggressive versus calm *	26.97	7.71	22.63	6.81	11.463	1	35	0.002	0.247
inhibited versus spontaneous	29.86	6.24	27.08	7.02	4.747	1	35	0.036	0.119
lonely versus secure	30.50	6.70	26.52	4.99	14.05	1	35	0.001	0.286
imbalanced feeling versus well-being *	57.80	14.5	45.36	13.73	24.213	1	35	0.001	0.409

* significant difference between female and male participants in pre-post difference according to GLM (ANOVA) – details see Appendix A.

**Table 5:** Mean differences in females regarding the subscales of the SDQ III before and after dog-assisted intervention.

Subscales SDQ III	Time 1		Time 2		F	df 1	df 2	*p*	η²
	M	SD	M	SD					
Math *	46.22	10.86	44.32	15.8	0.421	1	30	0.521	0.014
Verbal *	43.45	10.65	55.74	10.85	45.87	1	30	0.001	0.605
Academic *	46.29	10.36	48.03	12.48	0.579	1	30	0.453	0.019
Problem solving	48.64	11.31	56.77	7.12	8.673	1	30	0.006	0.224
Physical ability *	44.48	9.19	51.32	11.73	15.196	1	30	0.001	0.336
Same sex peer relations *	38.22	7.48	52.54	12.16	32.353	1	30	0.001	0.519
Opposite sex peer relations *	41.03	16.15	56.35	7.03	21.938	1	30	0.001	0.422
Parent relation	39.19	7.91	44.58	15.47	4.026	1	30	0.054	0.118
Spiritual values/religion *	57.22	8.89	45.29	12.54	14.401	1	30	0.001	0.324
Honesty/trustworthiness *	49.61	11.89	68.32	12.06	32.763	1	30	0.001	0.522
Emotional stability *	51.25	13.67	50.19	12.24	0.116	1	30	0.736	0.004
General esteem	54.19	10.69	69.93	11.41	35.587	1	30	0.001	0.543
Physical appearance	43.00	11.02	41.45	5.78	0.394	1	30	0.535	0.013

* significant difference between female and male participants in pre-post difference according to GLM (ANOVA)—details see Appendix A.

**Table 6:** Mean differences in males regarding the subscales of the SDQ III before and after dog-assisted intervention.

Subscales SDQ III	Time 1		Time 2		F	df 1	df 2	*p*	η²
	M	SD	M	SD					
Math *	43.49	17.45	47.79	14.82	10.533	1	48	0.002	0.180
Verbal *	53.67	11.01	59.45	10.79	32.628	1	48	0.001	0.405
Academic *	45.79	14.00	53.75	15.19	37.231	1	48	0.001	0.437
Problem solving	51.93	10.96	64.26	8.76	93.38	1	48	0.001	0.661
Physical ability *	51.83	15.38	53.73	14.34	3.078	1	48	0.086	0.060
Same sex peer relations *	52.00	10.77	58.97	10.38	39.333	1	48	0.001	0.450
Opposite sex peer relations *	53.42	10.67	56.95	11.39	8.788	1	48	0.005	0.155
Parent relation	46.97	15.36	48.28	16.08	0.833	1	48	0.366	0.017
Spiritual values/religion *	49.81	16.28	50.53	20.33	0.180	1	48	0.673	0.004
Honesty/trustworthiness *	66.4	9.99	74.36	8.14	46.417	1	48	0.001	0.492
Emotional stability *	51.95	12.64	63.36	2.24	63.615	1	48	0.001	0.570
General esteem	66.08	14.79	80.18	10.71	78.002	1	48	0.001	0.619
Physical appearance	49.89	11.84	51.53	10.35	1.9	1	48	0.174	0.038

* significant difference between female and male participants in pre-post difference according to GLM (ANOVA)—details see Appendix A.

## Appendix A

**Table A1:** Mean differences between males and females regarding the subscales of the SEE according to GLM (ANOVA)**.**

Subscales SEE	F	df 1	df 2	*p*	η²
accepting own emotions	6.285	1	79	0.014	0.074
emotional flooding	0.091	1	79	0.763	0.001
lack of emotions	5.809	1	79	0.018	0.068
somatic representation	0.627	1	79	0.431	0.008
imaginative representation	1.455	1	79	0.231	0.018
emotional regulation	9.954	1	79	0.002	0.112
self-control	5.480	1	79	0.022	0.065

**Table A2:** Mean differences between males and females regarding the subscales of the EMI-B according to GLM (ANOVA)**.**

Subscales EMI-B	F	df 1	df 2	*p*	η²
anxious versus free from fear	10.265	1	61	0.002	0.144
depressive versus happy	15.014	1	61	0.001	0.198
tired versus dynamic	8.776	1	61	0.004	0.126
aggressive versus calm	17.03	1	61	0.001	0.218
inhibited versus spontaneous	3.52	1	61	0.065	0.055
lonely versus secure	3.331	1	61	0.073	0.052
imbalanced feeling versus well-being	10.475	1	61	0.002	0.147

**Table A3:** Mean differences between males and females regarding the subscales of the SDQ III according to GLM (ANOVA).

Subscales SDQ III	F	df 1	df 2	*p*	η²
Math	4.705	1	78	0.033	0.057
Verbal	11.498	1	78	0.001	0.128
Academic	6.448	1	78	0.013	0.076
Problem solving	2.391	1	78	0.126	0.030
Physical ability	10.282	1	78	0.002	0.116
Same sex peer relations	7.385	1	78	0.008	0.086
Opposite sex peer relations	15.497	1	78	0.001	0.166
Parent relation	2.141	1	78	0.147	0.027
Spiritual values/religion	14.950	1	78	0.001	0.161
Honesty/trustworthiness	11.548	1	78	0.001	0.129
Emotional stability	16.200	1	78	0.001	0.172
General esteem	0.319	1	78	0.574	0.004
Physical appearance	0.298	1	78	0.587	0.004
